# Synthesis of ZSM-5/Siliceous Zeolite Composites for Improvement of Hydrophobic Adsorption of Volatile Organic Compounds

**DOI:** 10.3389/fchem.2019.00505

**Published:** 2019-07-16

**Authors:** Renna Li, Shijia Chong, Naveed Altaf, Yanshan Gao, Benoit Louis, Qiang Wang

**Affiliations:** ^1^College of Environmental Science and Engineering, Beijing Forestry University, Beijing, China; ^2^ICPEES - Institut de Chimie et Procédés pour l'Énergie, l'Environnement et la Santé, UMR 7515 CNRS - Université de Strasbourg, Strasbourg, France

**Keywords:** toluene adsorption, composite materials, ZSM-5, siliceous zeolite, hydrophobicity evaluation

## Abstract

In this research, we investigated the hydrophobicity and dynamic adsorption-desorption behaviors of volatile organic compounds (VOCs) by applying different optimized coating dosage (25, 50, and 75%) on designed novel ZSM-5/MCM-41 and ZSM-5/Silicalite-1 hierarchical composites. The relatively large specific surface area and pore volume of adsorbents ZSM-5/MCM-41 and ZSM-5/Silicalite-1 composites with excellent stability were affirmed by *ex-situ* XRD, FTIR, BET, SEM, and water contact angle analyses. Regarding, toluene adsorption-desorption investigation, ZSM-5/MCM-41 composite lead a longer stable toluene breakthrough time no matter under dry or 50% humid conditions. However, under different loading dosage condition, the breakthrough time of 75% coating ratio was the longest, which was 1.6 times as long as that of pure ZSM-5 under wet adsorption. Meanwhile, the complete elimination of toluene for ZSM-5/MCM-41-75% was done by largest desorption peak area and the lowest desorption temperature of 101.9°C, while, the largest contact angle of ZSM-5/MCM-41-75% was 17.0° higher than pure ZSM-5 zeolite. Therefore, we believe that the present hydrophobic sorbent will provide new insight with great research potential for removing low concentration of VOCs at industrial scale.

## Introduction

With industrialization, volatile organic compounds (VOCs), as the significant precursors of photochemical smog and secondary organic aerosol (SOA), have raised stringent environmental threat for many industrial processes. Generally, VOCs originate from a wide range of manmade sources (e.g., industrial processes, vehicle emissions, solvent manufacturing, gasoline evaporation, fuel coatings manufacturing) and in a gaseous state at atmospheric pressure of 133.322 Pa (Okada et al., 2012). VOCs were promulgated to be composed of more than 500 compounds with different properties and most of them were demonstrated to be with high toxicity (Lewis et al., 2000). Being as an important photochemical oxidant of atmospheric environment, some of VOCs act as nerve agent could cause neurological disturbance and cell cancerization, bringing a great threat to human health (Atkinson, 2000). Therefore, the VOCs pollutants removal has already been one of the most urgent research areas.

In previous studies, several VOCs abatement technologies were developed, including oxidation (Johnsen et al., 2016; Zhang et al., 2016), biological treatment (Chen et al., 2012; Cheng et al., 2016), condensation (Khan and Ghoshal, 2000), absorption (Darracq et al., 2010; Hariz et al., 2017) and adsorption (Zhang et al., 2017). However, constrain was found in each technology during practical and industrial application. Such as, operating expense for VOCs extirpation by oxidation technology is extravagant due to high reaction temperature and longer retention time. As for biological treatment, the removal efficiency of VOCs is low, and the persistence time is relatively long. Condensation and absorption technologies would escalate the operating cost due to the secondary treatment of scrubbing liquids and adsorbents. Moreover, compared to these techniques, adsorption is more applicable for removing low concentration of VOCs due to its advantages (i.e., high efficiency, easy operation and low system operating costs) (Kim and Ahn, 2012; Tefera et al., 2014).

It is well-recognized that removal efficiencies by adsorption depend on the types and properties of adsorbents. Many materials have been widely accounted in adsorption of VOCs, containing carbon materials [e.g., activated carbon (Li et al., 2011; Qian et al., 2015), graphene (Shen et al., 2015) and activated carbon fiber Baur et al., 2015], oxygen adsorbents [e.g., zeolite (Li and Yu, 2014), silica gel (Adjimi et al., 2014) and metallic oxide Castaño et al., 2017], and polymeric adsorbents [e.g., polymer adsorption resin Wang et al., 2014]. Especially, activated carbon and zeolite are enumerated as main adsorbent controlled VOCs pollutants by the United States Environmental Protection Agency (USEPA). Withal, activated carbon materials (e.g., activated carbon, surface modified carbon) were profoundly applied owing to their strong adsorption selectivity for VOCs. However, the existence of fire and safety threat, stomatal blockage, and insufficient regenerative capacity of activated carbon materials limited their practical application (Zhao et al., 1998). Among these, microporous zeolites are good candidates for adsorbents of VOCs due to their unique properties such as simple synthesis, high adsorption capacity, high thermal stability and so on.

In our previous study (Li et al., 2018), although microporous zeolites (such as Y, ZSM-5 and TS-1) revealed and elucidated that high adsorption capacity for toluene, serious hydrophilicity of zeolites limits their application. Accordingly, the synthesis and application of hydrophobic zeolites have been appealing intense attentions. Therein, all-silicon zeolites may provide an opportunity to prepare relative hydrophobic zeolite due to resist the influence of some water vapor because of its pure silicon-type material (despite it contains a little the silanol on the amorphous walls surface) (Serrano et al., 2004; Dou et al., 2011). Therefore, pure siliceous zeolites are loaded on microporous zeolite ZSM-5, which can not only form multilayer porous materials, but also improve the hydrophobicity of microporous zeolite. Few reported researches work about synthesis of ZSM-5/all-silicon zeolites (e.g., MCM-41, SiO_2_, and Silicalite-1) composites. Most of the modified materials were used for catalytic properties, but few reports on removal adsorption of VOCs (Li et al., 2016; Pan et al., 2018; Xian et al., 2018).

Here, we aimed to synthesize hierarchically porous ZSM-5/MCM-41 and ZSM-5/Silicalite-1 composites to improve the adsorption of microporous zeolite for toluene adsorption under humidity condition. To this end, ZSM-5/MCM-41 and ZSM-5/Silicalite-1 composites were synthesis and their adsorption properties for toluene under humidity condition were determined. Meanwhile, ZSM-5/MCM-41 and ZSM-5/Silicalite-1 composites were characterized by *ex-situ* manner to prove the advantages of multiple pores zeolites.

## Materials and Methods

### Materials

Cetyltrimethyl ammonium bromide (CTAB), sodium silicate (Na_2_SiO_3_·9H_2_O) and tetrapropylammonium hydroxide (TPAOH) were bought from China Pharmaceutical Group Chemical Reagents Co., Ltd., the sodium hydroxide (NaOH) was bought from Xilong Science Co., Ltd., sulphuric acid (H_2_SO_4_) and tetraethylorthosilicate (TEOS) were purchased from Beijing Chemical Works and Aladdin, respectively.

### Preparation of Zeolites and Compounds

Zeolite ZSM-5 was synthesized according to the previous study (Kustova et al., 2006). First, 0.3 g NaOH and 0.3 g NaAlO_2_ were dissolved in 9.8 g distilled water, then 19.4 g of TPAOH and 19.7 g TEOS were added into the above solution and stirred for 2 h at room temperature. The homogeneous gel was then transferred into a Teflon lined stainless steel autoclave and hydrothermal treated at 170°C for 72 h. Then, the product was cooled and centrifugation with deionized water until pH was 7. The product finally calcined at 550°C for 5 h. The Si/Al molar ratio of pure ZSM-5 was about 100.

A pure silica MCM-41 was synthesized by a hydrothermal treatment (Serrano et al., 2004). Initially, 2.4 g of CTAB and 0.48 g of NaOH were dissolved in 60 g deionized water. Thereafter, 14.75 g of TEOS was added into solution and the mixture was stirred for 5 h at room temperature. After that, the resultant solution was loaded in a Teflon lined stainless steel autoclave, and treated during 48 h under static conditions at 110°C. The obtained solid product was filtered, washed with distilled water. The calcination for removing the surfactant was performed at 550°C in air for 8 h.

ZSM-5/MCM-41 composite was synthesized according to the following steps (Witsarut and Sirirat, 2016). First, 4.25 g of CTAB was dissolved in 40 mL of deionized water, then 1.2 g of ZSM-5 was added to the solution with stirring for 24 h at 25°C to obtain slurry A. Solution B was obtained by dissolving 9.79 g of sodium silicate in 100 mL deionized water with pH was 11 adjusted by adding 6 M H_2_SO_4_. After stirring for several minutes, slurry A was dropwise added to solution B, the mixture solution was stirred for 2 h at room temperature. Then the gel solutions were transferred into a Teflon lined stainless steel autoclave and heated at 120°C for 72 h. Finally, the ZSM-5/MCM-41 compound was filtered, washed, dried, and calcined at 550°C for 6 h.

Synthesis of ZSM-5/Silicalite-1 composite was followed by previous research steps (Deng et al., 2014). Specifically, 1.22 g of TPAOH was solved into 30 mL of deionized water, then 3.0 g of ZSM-5 zeolite was added to the mixture solution with ultrasonic treatment for 30 min. Next, 2.14 g of TEOS was added drop by drop to above mixture with stirring for 3 h at 80°C. The mixture was heated in an autoclave at 180°C under static condition for 48 h. Finally, the composite was collected by centrifugation, then washing, drying and calcination at 550°C for 5 h. Synthesis of Silicalite-1 was the same as the above method, the only difference was removal of ZSM-5.

According to the above methods, the coating ratio of MCM-41 and Silicalite-1 were 50%. Composite materials with 25 and 75% MCM-41 or Silicalite-1 were synthesized by adjusting the dosage.

### Characterization

X-ray diffraction (XRD) analysis were measured on a Shimadzu XRD-7000 instrument in reflection mode with Cu Kα radiation. The diffraction patterns of low-angle and high-angle were obtained over the range of 1–8° and 5–50°, with a scanning rate of 1° min^−1^ and 5° min^−1^, respectively. The 2 theta step was 0.02°. Infrared spectroscopic (IR) analysis was carried out on a Bruker Vertex 70 spectrophotometer with a wavenumber resolution of ±4 cm^−1^ and scanning range from 4,000 to 400 cm^−1^, the number of scans used for the IR measurements was 32 times. Before test, the materials were blended with KBr and then pressed wafers. The nitrogen adsorption and desorption tests were conducted in Kubo-X1000 apparatus at 77 K. The specific surface area of samples was obtained by the linear of the Brunauer-Emmett-Teller (BET) equation, and the pore size distribution of materials was determined from the nitrogen physisorption isotherms using the T-plot method and the Barret-Joyner-Halenda (BJH) method. The surface morphology of products was measured using a scanning electron microscopy (SEM, Hitachi S-3400N II). Hydrophobicity of samples was evaluated through dynamic water contact angle analyses. The water contact angles of advancing and receding were gauged with a contact angle goniometer (Dataphysics OCA20) equipped with uEye digital camera. The syringe was filled with pure water, the droplet volume was 3 μL and the drop speed was 5 μL min^−1^. For accuracy, each sample was measured for 5 times.

### Measurement of Toluene Adsorption-Desorption Performance

Toluene adsorption capacity of samples was evaluated using a fixed-bed reactor equipped with a GC apparatus (Agilent 7890A). The front detector used for gas chromatograph was a flame ion detector (FID) and the rear detector was a thermal conductivity detector (TCD). For each test, approximately 100 mg pretreated sample was loaded into the stainless-steel reactor with inner diameter of 1 cm. The samples pretreatment was done in the vacuum oven at 110°C for overnight to remove impurities and moisture before adsorption. Consequently, the testing condition is as follows: 400 ppm toluene and 20% O_2_, balanced by Ar with a total flow rate of 100 mL/min. For the wet condition, by winding heating belt outside the water vapor device and reaction tube to control the relative humidity of 50%. The desorption test was conducted from 40 to 200°C with a heating rate of 2°C min^−1^ by blowing 40 mL min^−1^ Ar. The thermal stability of adsorbed toluene was evaluated by a temperature programmed desorption (TPD) test. First, the sample was exposed to 2,000 ppm toluene with a flow rate of 40 mL min^−1^ at 40°C for 30 min. The toluene-TPD test was then implemented by heating the sample with a ramp of 10°C min^−1^ from 40 to 600°C. The calculation of the saturated adsorption of toluene is according to the following formula (1):

(1) $$\begin{array}{r} q=\frac{F^{*}C_{0}^{*}10^{-9}}{W}\left[t_{s}-\int_{0}^{t_{s}}\frac{C_{t}}{C_{0}}d_{t}\right] \end{array}$$

where q (mg/g)—adsorption capacity of toluene,

F (mL/min)—gas flow velocity,

C_0_ (mg/m^3^)—initial concentration of the intake,

C_t_ (mg/m^3^)—the concentration of gas at t minutes,

W (g)—the amount of adsorbent,

t (min)—the adsorption time,

t_s_ (min)—the time of adsorption saturation.

## Results And Discussion

### Characterization of ZSM-5/Siliceous Zeolite Composites

**Figure 1:**
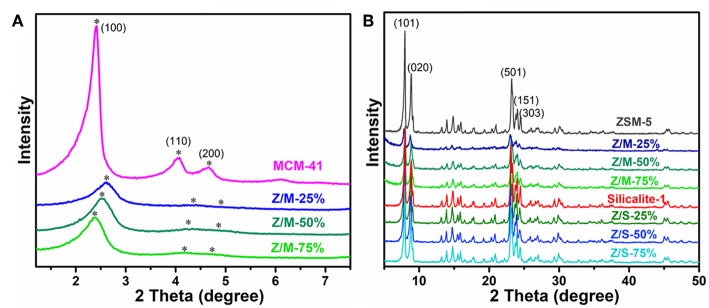
XRD patterns of ZSM-5/siliceous zeolite composites at **(A)** low-angle and **(B)** high-angle.

The low-angle and high-angle XRD patterns of ZSM-5, MCM-41, Silicalite-1, ZSM-5/MCM-41 composite and ZSM-5/Silicalite-1 composite are shown in Figure 1. As per Figure 1A of the low-angle XRD patterns, ZSM-5/MCM-41 composites exhibited the characteristic peak of (100), which belonged to the hexagonal mesopore structure of MCM-41, and the intensity of the peaks increased with the increasing of MCM-41 loading. Noteworthy, in comparison with pure MCM-41, the (100) diffraction peak of composites deviated to high-angle, which was more deviation as the load decreases and the diffraction peak of (200) became quite weak. This was due to the certain influence of alkali treatment on aggregated micelles during hydrothermal process and subsequent formation of mesopores (Sang et al., 2013). The result illustrates that the part of ZSM-5 particulates were disintegrated into Si-Al nanoclusters then forming the hexagonal mesopore structure owing to the presence of CTAB templates (Tanaka et al., 2008). The high-angle XRD patterns of ZSM-5, Silicalite-1 and all the compounds appeared the diffraction peaks at 2θ = 7.9°, 8.7°, 23.1°, 23.9°, and 24.4°, respectively, which corresponding to the reflections of (101), (020), (501), (151), and (303) planes (Figure 1B). The result demonstrates that all samples present a MFI-type zeolite structure (Sang et al., 2013). There was no presence of impurity phases in XRD curves, indicating the successful synthesis of ZSM-5/siliceous zeolite composites.

**Figure 2:**
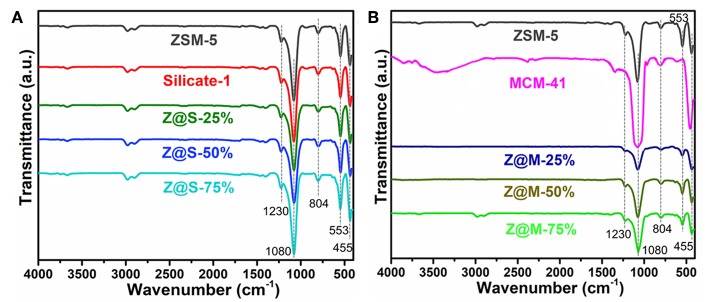
IR spectra of **(A)** ZSM-5/MCM-41 (Z/M) and **(B)** ZSM-5/Silicalite-1 (Z/S) composites.

IR spectra of ZSM-5/MCM-41 and ZSM-5/Silicalite-1 composites with different siliceous zeolite loading are shown in Figure 2. The strong band appeared at 1,080 cm^−1^ with a little shoulder at 1,230 cm^−1^ was assigned to the T–O–T (T was Si or Al atom) asymmetric stretching mode. Consequently, the relatively weak band at 804 cm^−1^ and the strong band at 455 cm^−1^ was owing to the corresponding T–O–T symmetric stretching mode with plane bending character and the T–O–T rocking mode, respectively. Thus, the weaker absorption bond in region 700–500 cm^−1^ and the quite strong band at 554 cm^−1^ were typical MFI structural units (Dutta et al., 1991; Li et al., 2013). These IR spectra indicated that all compounds consistent with the above XRD results Figure 1, which contained the primary or secondary structure of ZSM-5 zeolite.

**Figure 3:**
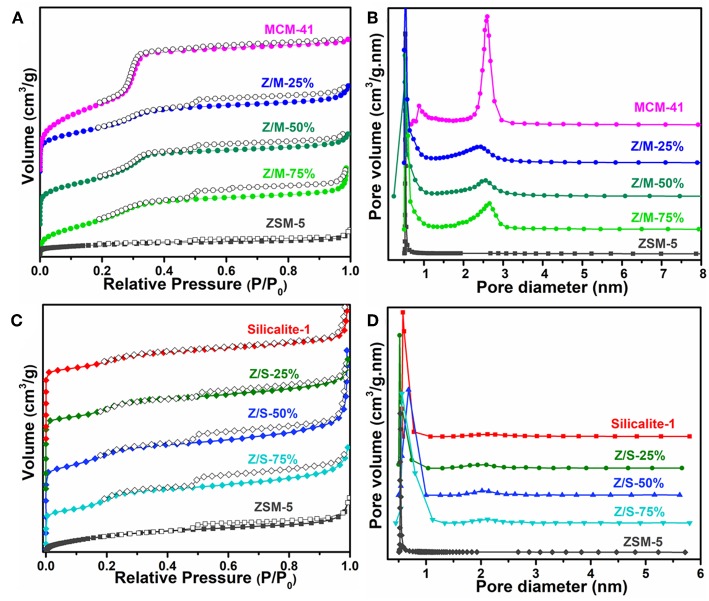
N_2_ adsorption–desorption isotherms of **(A)** MCM-41, ZSM-5, ZSM-5/MCM-41 with different dosage and **(C)** Silicalite-1, ZSM-5/Silicalite-1 with different dosage. Pore size distribution curves of **(B)** MCM-41, ZSM-5, Slicalite-1 and **(D)** all ZSM-5/siliceous zeolite composites.

The N_2_ adsorption-desorption isotherms and pore distribution of ZSM-5, MCM-41, Silicalite-1 zeolite and different composites are depicted in Figure 2. Moreover, it can be explicated from Figure 3A, comparing with ZSM-5, the isotherms of ZSM-5/MCM-41 compounds were similar to type I in pressure range P/P_0_ < 0.05, the adsorption capacity of N_2_ was below 140 cm^3^/g, which was the amount of the microporous filling volume. However, the N_2_ adsorption–desorption isotherms of ZSM-5/MCM-41 were belong to those of type IV in the pressure range P/P_0_ > 0.05, which is typical for mesoporous zeolite and it illustrated the existence of mesopores. As per Figure 3B, there were two kinds of pore size distribution in all composites except microporous zeolite ZSM-5. The average diameter of the micropores was around 0.53 nm, and mesopores with pore diameters of 2–3.5 nm. However, as shown in Figure 3C, it can be found that the N_2_ adsorption isotherms of ZSM-5/Silicalite-1 were belong to type I as a whole, but there is a small hysteresis loop at the pressure range of 0.5–0.9, which indicates the existence of mesopores in the composites. Such result was consistent with the pore size distribution in Figure 3D.

The BET parameters of pure ZSM-5, MCM-41, Slicalite-1, and all ZSM-5/siliceous zeolite composites were listed in Table 1, in which the specific surface area (SSA) of ZSM-5/MCM-41 composite zeolites with different dosage obey the larger specific surface area with increasing dosage ratio of MCM-41, however, ZSM-5/Silicalite-1 composites had the opposite order. Moreover, the same table also reflected that there were double-pore in all the ZSM-5/siliceous zeolite composites, but in fact, the mesopore of ZSM-5/Silicalite-1 composites was very weak, and it was still dominated by existing micropores, which was consistent with the information in Figure 3.

**Table 1:** BET parameters of pure ZSM-5, MCM-41, Slicalite-1, and all ZSM-5/siliceous zeolite composites.

**Samples**	**S_**BET**_ (m^**2**^/g)**	**Pore size (nm)**	**V_**total**_ (cm^**3**^/g)**	**V_**micro**_ (cm^**3**^/g)**	**V_**meso**_ (cm^**3**^/g)**
ZSM-5	377	0.53	0.173	0.137	0.012
MCM-41	896	2.60	0.714	0.179	0.534
Z/M-25%	593	0.53, 2.44	0.475	0.261	0.210
Z/M-50%	645	0.52, 2.56	0.499	0.267	0.229
Z/M-75%	706	0.52, 2.66	0.563	0.288	0.273
Silicalite-1	361	0.60, 2.12	0.287	0.169	0.117
Z/S-25%	466	0.52, 2.10	0.412	0.211	0.198
Z/S-50%	439	0.68, 2.02	0.301	0.203	0.097
Z/S-75%	397	0.56, 2.12	0.276	0.187	0.088

**Figure 4:**
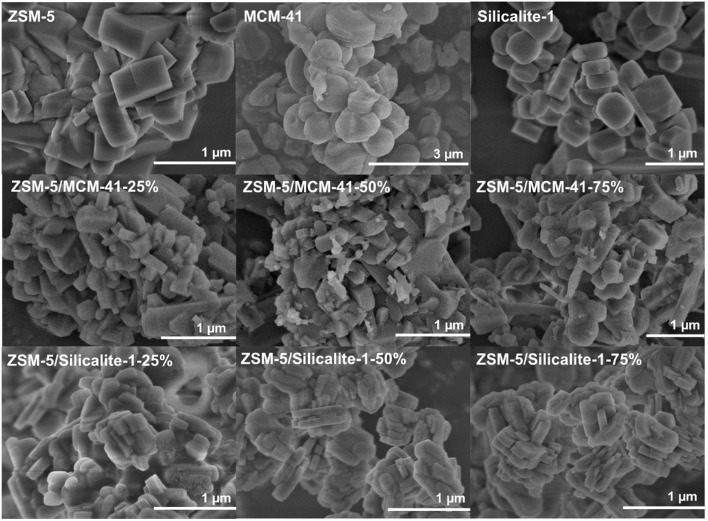
SEM images of MCM-41, ZSM-5, Slicalite-1, and all ZSM-5/siliceous zeolite composites.

Moreover, Figure 4 demonstrated the SEM images of MCM-41, ZSM-5, and the corresponding composite ZSM-5/MCM-41, ZSM-5/Silicalite-1 with different dosage. Pure MCM-41 and ZSM-5 zeolite illustrated spherical particles and regular cubic particles, respectively. For ZSM-5/MCM-41 compounds, compared with the uniform morphology of pure ZSM-5, a lot of ZSM-5 particles were disintegrated and became irregular due to desilication phenomenon in the framework of ZSM-5 under basic conditions then destruction of silicon. It is worth noting that MCM-41 particles cannot be coated on the external surface of ZSM-5 because of the size of the particles, while it can only coexist with ZSM-5 to form microporous-mesoporous multilayer structure materials (Shen et al., 2018). With Silicalite-1 coating, the ZSM-5/silicalite-1 composites retain the big granular morphology but with the rough surfaces and the smooth corners, also it was apparent that there was a contiguous Silicalite-1 layer on the outer surface of ZSM-5 crystals, that the coating became thicker with the increase of dosage.

### Toluene Adsorption Properties of Materials

**Figure 5:**
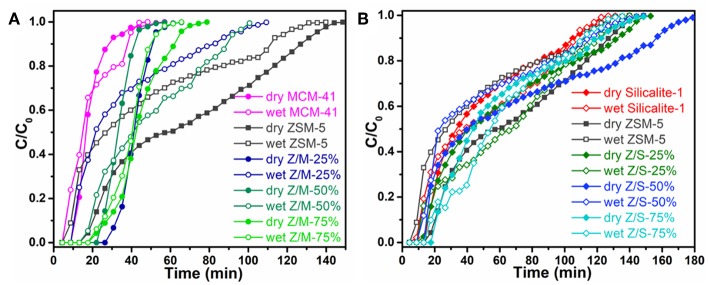
Toluene breakthrough curve of **(A)** ZSM-5/MCM-41 and **(B)** ZSM-5/Silicalite-1 composites under dry and wet conditions.

The effect of siliceous zeolite coating for the toluene adsorption was systematically investigated under both dry and wet conditions. The toluene breakthrough curves are shown in Figure 5, the calculation of saturated adsorption capacity is based above Equation (1). Figure 5A clearly show that the breakthrough time of zeolite/MCM-41 composites became longer than that of parent zeolites no matter under dry or wet conditions. It was reported that a longer breakthrough time indicated a better adsorption capacity for a constant concentration. Under humidity conditions, the breakthrough curves of all microporous composites were greatly tilt, which was due to the adsorption sites occupied by water molecules lead the slow diffusion of toluene and longer saturation time. Figure 5B shows that all the adsorption curves take a quite long time from breakthrough to saturation, illustrating that a big mass transfer resistance. This was due to the main narrow micropore entrance within the microporous zeolites can lead to a low diffusion rate (Kim and Ahn, 2012). Nevertheless, it also can be seen from the Figure 5 that all the ZSM-5/Silicalite-1 complexes have longer breakthrough time than ZSM-5 under 50% relative humidity condition.

Table 2 elucidates the toluene adsorption properties of various samples under dry and wet conditions, which were obtained from the breakthrough curves in Figure 5. As a whole, ZSM-5/MCM-41 compounds with different dosage gave a longer breakthrough time for toluene, while presented a lower adsorption capacity due to the lower saturation time. From Table 1, the specific surface area of ZSM-5/MCM-41 was also larger than major microporous zeolites. The adsorption capacity of microporous adsorbents was mainly affected by the specific surface area and pore volume, this result was consistent with gravimetric adsorption isotherm data (Huang et al., 2002). However, the longest breakthrough time decreased with the increasing of MCM-41 due to the poor adsorption property (only 9.9 under dry condition) of MCM-41. On the contrary, the adsorption properties increased with the increasing of MCM-41 or silicalite-1 under wet condition, this is mainly because of the better hydrophobic property of MCM-41 and silicalite-1. Both ZSM-5/MCM-41 and ZSM-5/Silicalite-1 composite materials present the best toluene adsorption property with 75% MCM-41 or silicalite-1 loading. Under 50% humidity conditions, the breakthrough time of ZSM-5/MCM-41-75% and ZSM-5/Silicalite-1-75% was 1.6 times and 1.2 times longer than that of pure ZSM-5, respectively. In most cases, the =Si-OH groups on the surfaces of silica-based materials act as the adsorption sites for various VOCs molecules. The toluene adsorption by siliceous zeolite act through weak π-system hydrogen bonding with silanols on outer surface (Kosuge et al., 2007).

**Table 2:** The toluene adsorption properties of MCM-41, ZSM-5, Slicalite-1 and all ZSM-5/siliceous zeolite composites with different dosage.

**Samples**	**Dry/Wet** **Breakthrough time (min)**	**Dry/Wet** **Saturation time(min)**	**Dry/Wet** **Saturated adsorption capacity (mg/g)**
ZSM-5	18.6/7.1	135.5/120.1	111.1/73.6
MCM-41	9.9/5.4	37.2/39.6	31.1/30.5
Z/M-25%	28.2/9.7	52.6/91.9	71.5/58.7
Z/M-50%	24.0/17.6	42.9/92.8	58.0/78.9
Z/M-75%	23.1/18.5	65.7/53.6	75.9/64.1
Silicalite-1	14.5/9.6	114.6/114.8	78.4/85.1
Z/S-25%	14.0/9.4	141.8/128.8	97.1/104.1
Z/S-50%	14.2/10.7	165.8/126.8	108.9/79.4
Z/S-75%	19.5/15.5	131.2/124.5	96.0/100.6

### Toluene-TPD of ZSM-5/Siliceous Compounds

**Figure 6:**
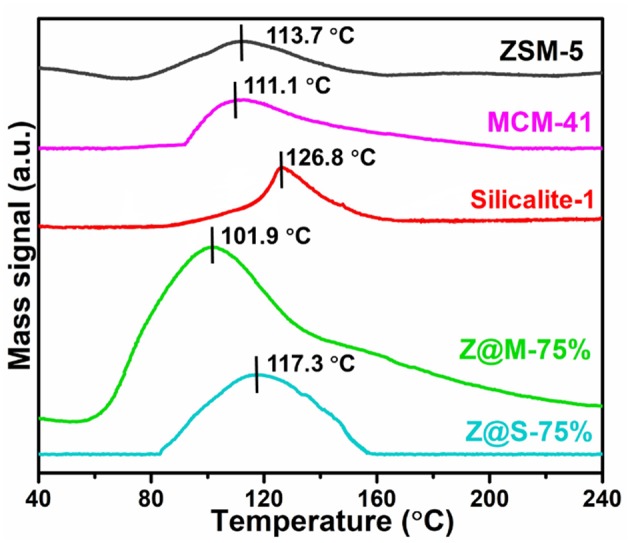
The toluene-TPD results for MCM-41, ZSM-5, Slicalite-1 and ZSM-5/siliceous zeolite composites with 75% dosage.

According to toluene-TPD test, toluene acting directly with the adsorption sites are desorbed by rising of the temperature. The desorption temperature is directly related to the intensity of adsorption adsorbents-adsorbates (Serrano et al., 2004). Figure 6 compares the toluene-TPD curve for three kinds of pure zeolites and two ZSM-5/siliceous-75% composites. The toluene-TPD curves of all adsorbents show just one desorption peak, indicating that there was only one type of adsorption site. In fact, the main desorption temperature of ZSM-5/MCM-41-75% was 101.9°C, which had the largest desorption peak area and the lowest desorption temperature, indicating that ZSM-5/MCM-41-75% was the best adsorbent among all the ZSM-5/siliceous composites. However, we did Ar-TPD and desorption test of ZSM-5/MCM-41-75%. Compare the curves of toluene-TPD and Ar-TPD, it is obvious that there was no peak for Ar-TPD (Figure S1A). Besides, one cyclic adsorption-desorption test was done by GC as well (Figure S1B). The result showed that there was no other desorption peak below 250°C except the one around 100°C, which was consistent with toluene-TPD result (101.9°C). Therefore, it can be confirmed that the peak around 101.9°C represent toluene. The desorption temperature results also indicated that 180°C was enough for toluene desorption for all the adsorbents. On the basis of TPD analysis, the cycling stability of ZSM-5 and ZSM-5/MCM-41-75% for toluene adsorption was studied, and 180°C was chosen as the desorption temperature. The experimental results show that both the materials have good cycling stability (Figure S2).

### Hydrophobicity Evaluation

**Figure 7:**
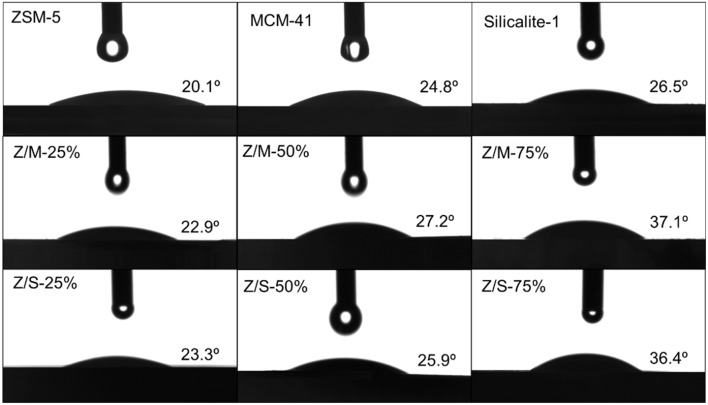
Contact angle measurement of MCM-41, ZSM-5, Slicalite-1, and all ZSM-5/siliceous zeolite composites.

Water contact angle can show hydrophilic or hydrophobic characteristics of the external surfaces directly. The images of the water drop on MCM-41, ZSM-5, Slicalite-1 and all ZSM-5/siliceous zeolite composites surfaces are shown in Figure 7. The contact angles of ZSM-5/MCM-41-25%, ZSM-5/MCM-41-50% and ZSM-5/MCM-41-75% were 22.9°, 27.2°, and 37.1°, respectively while the contract angles of ZSM-5/Silicalite-1-25%, ZSM-5/Silicalite-1-50% and ZSM-5/Silicalite-1-75% were 23.3, 25.9, and 36.4°, respectively. Out of all the contact angle measured, ZSM-5 zeolite displayed the lowest contact angle (20.1°) due to its hydrophilic properties. When ZSM-5 zeolite coated with siliceous MCM-41 and Silicalite-1, the compounds showed the obvious improvement hydrophobicity with higher water contact angles than that of ZSM-5. In addition, ZSM-5/MCM-41-75% and ZSM-5/Silicalite-1-75% showed the largest contact angle loading 75% dosage both composites, which was 37.1 and 36.4°, respectively. These results are in agreement with the previous results of toluene adsorption under wet condition. The higher hydrophobicity of composites with 75% dosage can be attributed to the combination of multiple scale surface roughness (Bernardoni and Fadeev, 2011). According to the SEM images (Figure 4), it is obvious that the surface of the ZSM-5/MCM41-75% and ZSM-5/Silicalite-1-75% composites was more roughness than that of pure zeolite. So the contact angle was larger than pure zeolite.

According to previous studies, microporous zeolite can be significantly improved hydrophobicity with coated all-siliceous zeolite, the other mechanism of hydrophobicity was that the most stable of water molecules was combined to surface cations, next by hydrogen linked to coordinated water or silanol groups bonded to dissociative water. Therefore, surface cations or Si-OH groups can make water molecules stay on the pore wall surface. However, free water weakly interacts with siliceous pore surface, because water molecules form amorphous groups through limited intermolecular hydrogen bonding (Cheng and Reinhard, 2006).

## Conclusions

The hydrophobicity and dynamic toluene adsorption-desorption behaviors were investigated for ZSM-5/MCM-41 and ZSM-5/Silicalite-1 hierarchical composites with different coating dosage, all of which had distinct characterization. Moreover, the crystal structure of ZSM-5/MCM-41 composites were somewhat destruction due to alkali treatment, which influence the formation of mesopores during hydrothermal process, and the existence of template destroyed orderliness of ZSM-5. On the contrary, ZSM-5/Silicalite-1 compounds showed better stability. For the study of toluene adsorption-desorption, the relatively large specific surface area and pore volume of ZSM-5/MCM-41 composite presented a longer toluene breakthrough time no matter in dry or 50% humid conditions. Under different loading dosage, the breakthrough time of 75% coating ratio was the longest, which was 1.6 times as long as that of pure ZSM-5 under wet adsorption. The largest contact angle of ZSM-5/MCM-41-75% was 17.0° higher than pure ZSM-5 zeolite. It was noteworthy, the reason for improvement of hydrophobic adsorption by coating siliceous zeolite was due to multiple scale surface roughness and free water form amorphous groups through finite intermolecular hydrogen bonds, so that water molecules interact weakly with the porous surface of siliceous materials.

## Data Availability

All datasets generated for this study are included in the manuscript/Supplementary Files.

## Author Contributions

RL and SC involved in the sample synthesis and performance test and wrote the draft manuscript. YG revised the manuscript and analyzed the experimental result. NA revised the manuscript. QW and BL conceived the project. All authors contributed to manuscript preparation.

### Conflict of Interest Statement

The authors declare that the research was conducted in the absence of any commercial or financial relationships that could be construed as a potential conflict of interest.
