# Supplementary material for: Synthesis of ZSM-5/Siliceous Zeolite Composites for Improvement of Hydrophobic Adsorption of Volatile Organic Compounds
Source: Front Chem. 2019 Jul 16;7:505. doi: 10.3389/fchem.2019.00505 (PMC6647869; doi:10.3389/fchem.2019.00505)
Supplement: Supplementary file 1 [file Data_Sheet_1.pdf]

# Supporting information

# Synthesis of ZSM-5/Siliceous Zeolite Composites for Improvement of Hydrophobic Adsorption of Volatile Organic Compounds

Renna Li[1], Shijia Chong[1], Naveed Altaf[1], Yanshan Gao[1,*], Benoit Louis[2], Qiang Wang[1,*]

[1] College of Environmental Science and Engineering, Beijing Forestry University, 35 Qinghua East Road, Haidian District, Beijing 100083, P. R. China.

[2] ICPEES - Institut de Chimie et Procédés pour l'Énergie, l'Environnement et la Santé, UMR 7515 CNRS - Université de Strasbourg, 25 rue Becquerel, 67087 Strasbourg cedex 2, France.

Corresponding author:

Assistant Professor Yanshan Gao,

Email: yanshan_gao@bjfu.edu.cn

Tel.: +86-15801531118

Professor Qiang Wang,

E-mail: qiang.wang.ox@gmail.com; qiangwang@bjfu.edu.cn

Tel: +86-13699130626

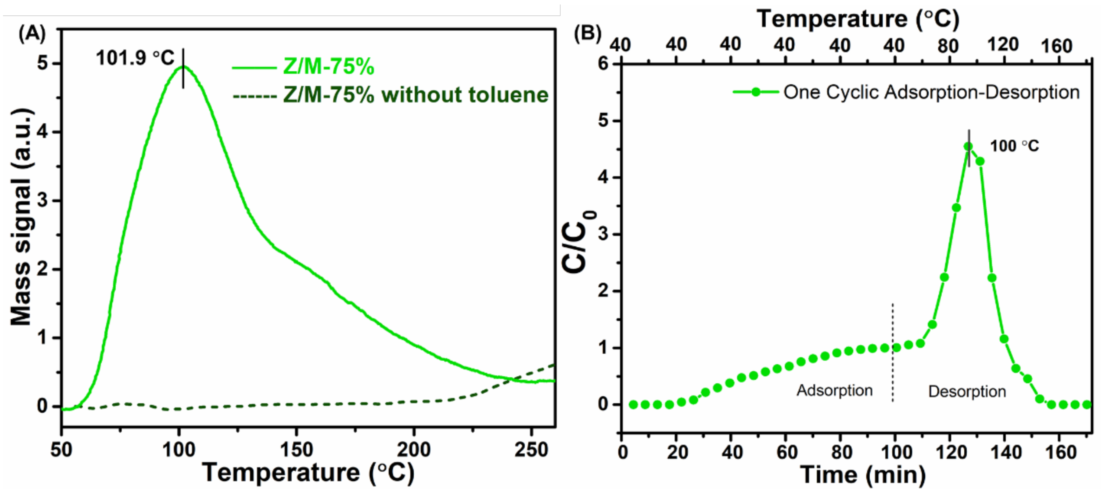


FIGURE S1 | (A) Toluene-TPD and Ar-TPD, (B) one cyclic adsorption-desorption of ZSM-5/MCM-41-75%.

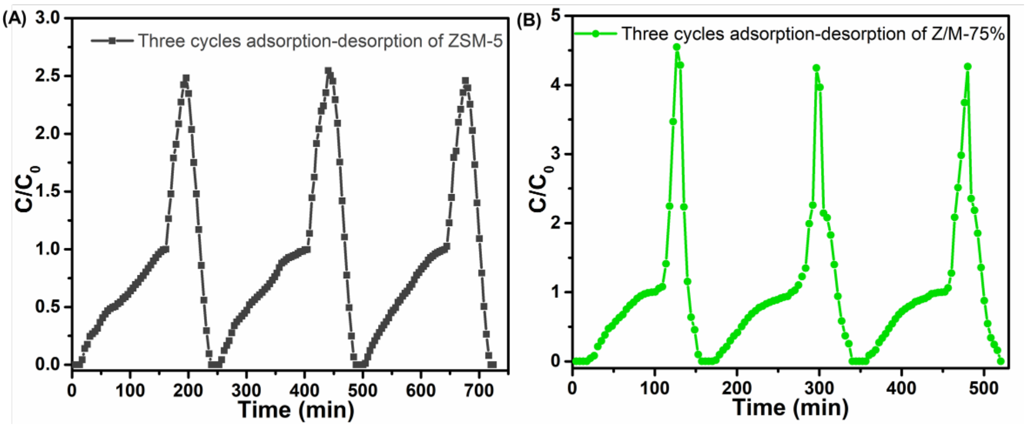


FIGURE S2 | Cycling stability of (A) ZSM-5 and (B) ZSM-5/MCM-41-75% samples.
